# Identification of key genes of anti-programmed death ligand 1 for meningioma immunotherapy by bioinformatic analysis

**DOI:** 10.1007/s12032-022-01869-8

**Published:** 2022-12-20

**Authors:** Lijian Zhang, Luxuan Wang, Yanli Tan, Chunhui Li, Chuan Fang

**Affiliations:** 1 Department of Neurosurgery, Affiliated Hospital of Hebei University, Hebei University, Baoding City, China; 2 Postdoctoral Research Station of Neurosurgery, Affiliated Hospital of Hebei University, Hebei University, Baoding City, China; 3 Hebei Key Laboratory of Precise Diagnosis and Treatment of Glioma, Baoding City, China; 4 Department of Neurological Examination, Affiliated Hospital of Hebei University, Hebei University, Baoding City, China; 5 Department of Pathology, Affiliated Hospital of Hebei University, Hebei University, Baoding City, China

**Keywords:** Meningioma, Bioinformatics, miR-155-5p, Immunotherapy, PD-L1

## Abstract

Meningioma is one of the most common primary tumors in the central nervous system (CNS). A deeper understanding of its molecular characterization could provide potential therapeutic targets to reduce recurrence. In this study, we attempted to identify specific gene mutations in meningioma for immunotherapy. One GSE43290 dataset was obtained from the Gene Expression Omnibus (GEO) database to find differentially expressed genes (DEGs) between meningioma tissues and normal meninges. In total, 420 DEGs were identified, including 15 up-regulated and 405 down-regulated genes. Functional enrichment analysis showed that these DEGs were mainly enriched in PI3K-Akt signaling pathway, Focal adhesion, and MAPK signaling pathway. We identified 20 hub genes by protein–protein interaction (PPI) analysis. Among the hub genes, the expression of FLT1, CXCL8, JUN, THBS1, FECAM1, CD34, and FGF13 were negatively correlated with Programmed Death Ligand-1 (PD-L1). Additionally, the expression of those genes was co-regulated by miR‐155‐5p. The findings suggest that miR-155-5p play an important role in the pathogenesis of meningioma and may represent potential therapeutic targets for its anti-PD-L1 immunotherapy.

**Supplementary Information:**

The online version contains supplementary material available at 10.1007/s12032-022-01869-8.

## Introduction

Meningioma is one of the most frequently diagnosed primary brain tumor, comprising approximately 36% of all brain tumors [1]. The origin of meningioma tumor is known as the arachnoid cells of the meninges. Based on their histopathologic features, the World Health Organization (WHO) classified them into 3 categories: benign (grade I), atypical (grade II), and anaplastic (grade III) [2]. In clinical settings, the standard therapies for meningiomas include surgery and/or radiation therapy. For some inoperable or incompletely operable grade II and III tumors, the optimal therapies are not well elucidated [3]. The emerging evidence demonstrated the importance of their molecular features for screening therapeutic targets and prognostic prediction [4]. Thus, a deeper understanding of the molecular alterations in meningioma could help improve clinical decision-making.

Recent genetic studies have identified several mutations that strongly correlated to the subtype, location, and growth rate, suggesting molecular profile might be more suitable for tumor classification [5]. Moreover, their molecular features could guide prediction and therapeutics and personalized and targeted therapies [6]. Monosomy 22 sequences and neurofibromatosis type 2 (NF2) are the most well-known genetic alterations founded in meningiomas [7, 8]. Recently, more genetic alterations and the signaling pathways were identified, such as mutations in TNF receptor associated factor 7 (TRAF7), AKT1, KLF4, and SMO, etc. [9–11]. Based on those above gene mutations, various targeted therapies have been trailed especially for patients with recurrent meningiomas. For example, Neurofibromatosis type 2 (NF2) is the first mutation identified in meningioma, which could be found in almost 50% of sporadic meningiomas [12]. Thus, novel therapies targeting NF2 such as focal adhesion kinase (FAK) inhibitors were developed [13, 14]. Nowadays, the translation of genomic knowledge into clinical management remains a challenge to scientists and neurosurgeons. Thus, a better understanding of their molecular landscapes could provide a tremendous opportunity to leverage and explore improved therapeutic strategies for meningiomas.

The developments in the field of genomics, proteomics, and metabolomics provide novel and deeper insights into the pathogenesis of meningiomas, as well as discover prospects for developing suitable and targeted interventions [15, 16]. By identification DEGs between meningioma tumors and normal meninges, we aimed to provide more information about the microenvironmental influence on meningioma development. Our study may contribute to screening potential therapeutic targets for meningioma.

## Method and material

### Microarray data

One dataset was obtained from the National Center of Biotechnology Information (NCBI) GEO database (https://www.ncbi.nlm.nih.gov/geo/). GSE43290 dataset was used in the present study, which included 47 tumor samples from meningioma patients and 4 normal meninges from healthy individuals [17].

### Data processing

The expression profiles of DEGs were obtained with GEO2R (http://www.ncbi.nlm.nih.gov/geo/geo2r/) [18]. Genes with |log2FC|> 2 and *P* value < 0.05 were selected as DEGs.

### Functional enrichment analysis of DEGs

The Gene Ontology (GO) and Kyoto Encyclopedia of Genes and Genomes (KEGG) enrichment analyses were performed with the Database for Annotation, Visualization, and Integrated Discovery (DAVID) database (https://david.ncifcrf.gov/tools.jsp). In the Gene Ontology (GO) database, gene functions are categorized into: cellular component (CC), biological processes (BP), and molecular functions (MF).

### Functional protein–protein interaction (PPI) analysis and hub-gene selection

The PPI network was performed by using the Search Tool for the Retrieval of Interacting Genes (STRING) database (http://string-db.org). The Cytoscape was used to visualize the PPI network (http://www.cytoscape.org/) (V3.7.2). Then, the Molecular Complex Detection (MCODE) in the Cytoscape software was used to obtain the modules within the PPI network.

### Analyzes of immune infiltration

The estimation of immune cell proportions was conducted by using the CIBERSORT web portal (http://CIBERSORT.stanford.edu/). CIBERSORT filters data with *P* value < 0.05. We obtained 22 types of immune cells. Then, we calculated the percentage of each immune cell type. Then, we analyzed the relationship between PD-L1 expression and the tumor-infiltrating immune cells. The results of immune infiltration were visualized by R packages (The R Project for Statistical Computing, version 4.1.0) [19].

### Correlation analysis between hub genes and PD-L1

The top 20 hub genes and their correlation with PD-L1 expression was analyzed and plotted on the GEPIA website (http://gepia2021.cancer-pku.cn/correlation.html).

### Transcription factor (TF)-miRNA coregulatory network

Interactions for TF-miRNA coregulatory were collected from the RegNetwork repository which assists to detect miRNAs and regulatory TFs that regulate DEGs of interest at the post-transcriptional and transcriptional levels. TF-miRNA coregulatory network was visualized using NetworkAnalyst (https://www.networkanalyst.ca/) (v2019) [20].

## Results

### DEGs identification

**Fig. 1:**
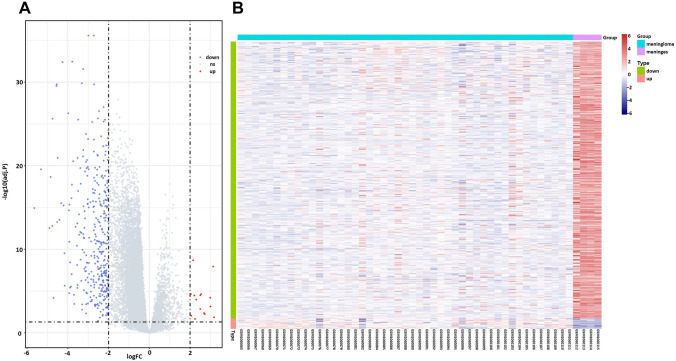
The overview of DEGs’ expression profile between meningioma tumors tissue and normal meninges. **A** The volcano map of DEGs in GSE43290. X: log2FC; Y: − log10 (*P* value). Blue represents down-regulating genes; red represents up-regulating genes. **B** Heatmap of DEGs in GSE43290. X: sample; Y: genes. Red represents high expression; blue represents low expression

In order to screen the DEGs that potentially participated in meningioma formation, DEGs analysis was performed between meningioma tumor tissue and normal meninges. In this study, we obtained 420 DEGs, including 15 up-regulating and 405 down-regulating (Supplementary Table 1). The expression profiles of those 420 DEGs were illustrated as volcano plot and a heatmap in Fig. 1A, B.

### Functional enrichment analysis for DEGs

**Fig. 2:**
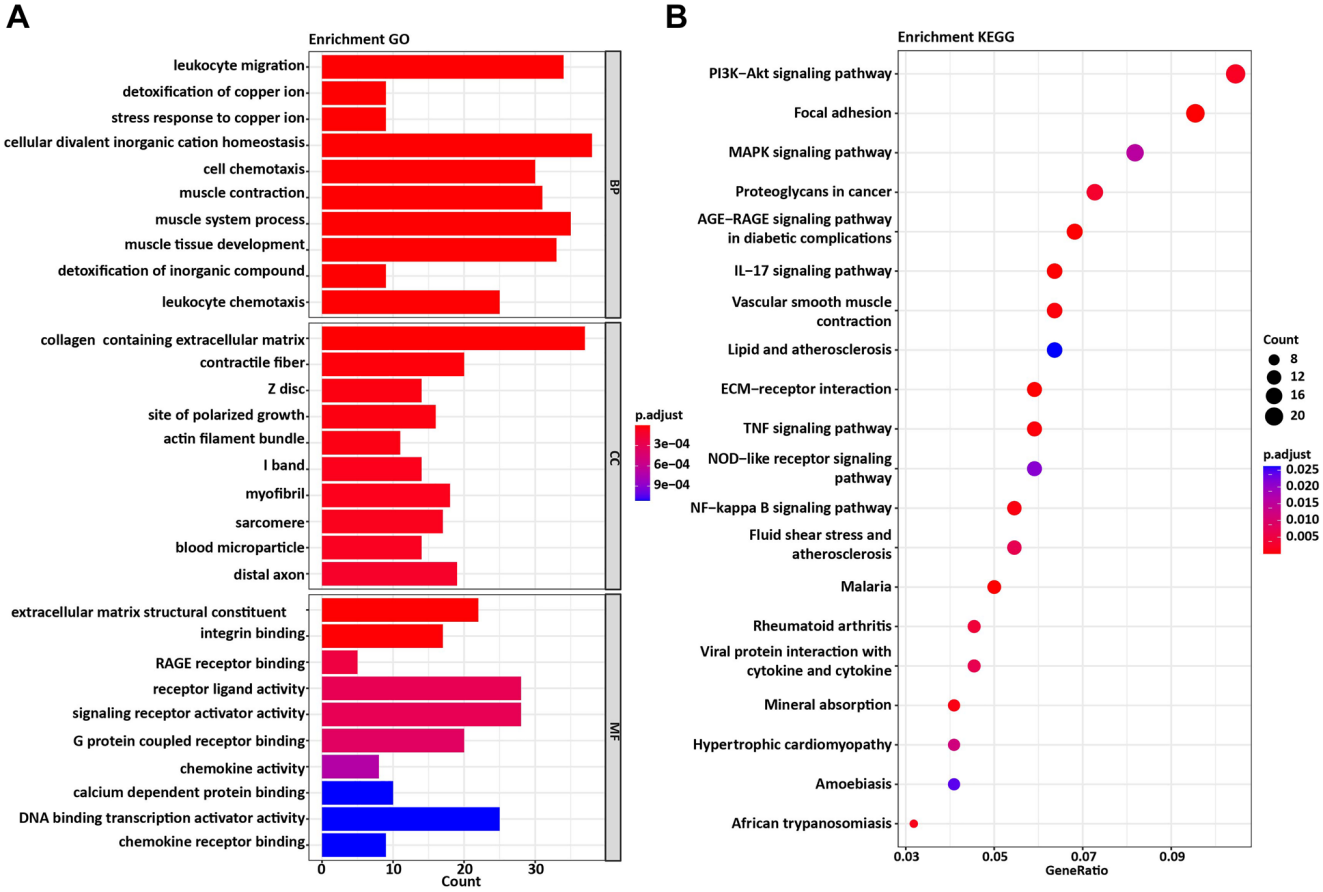
GO enrichment and KEGG pathway analysis of DEGs. **A** GO categories of MF, CC, and BP. **B** KEGG pathway analysis of the DEGs

To have a better understanding of how those DEGs participated in the pathologies of meningiomas, GO and KEGG enrichment analyses were performed. Detailed results of GO enrichment analysis are shown in Table 1. The most noteworthy enriched CC terms were collagen-containing extracellular matrix, neuronal cell body, and the actin cytoskeleton. The most noteworthy enriched BP terms were cellular divalent inorganic cation homeostasis, muscle system process, and leukocyte migration. The most noteworthy enriched MF terms were receptor ligand activity, signaling receptor activator activity, DNA-binding transcription activator activity, and RNA polymerase II-specific (Fig. 2A). Detailed results of the KEGG enrichment analysis are shown in Table 2. In KEGG pathway analysis, DEGs were dominant enriched in pathways in PI3K-Akt signaling pathway, Focal adhesion, and MAPK signaling pathway, etc. (Fig. 2B).

**Table 1:** Gene ontology (GO) functional enrichment analysis for the differentially expressed genes (DEGs)

Term	Category	*P* value	Genes name
*Cellular component*			
Collagen-containing extracellular matrix	GO:0,062,023	1.19365E-13	MMRN2, TNN, COL14A1, GPC5, APOA1, ADAMTS9, COL4A3, ACAN, TNC, ADAMTS1, S100A9, S100A4, ICAM1, ANGPT1, VWF, THBS1, ANGPTL7, S100A8, MATN4, DPT, LAMA2, THBS4, IGFBP7, DCN, AGT, COL2A1, CXCL12, AEBP1, PRELP, SERPINA3, SRPX, LUM, COL9A3, COL4A2, ASPN, MXRA5,, FBLN1
Contractile fiber	GO:0,043,292	9.17E-08	HOMER1, MYH11, FHOD3, LDB3, FHL5, CASQ2, ACTA2, PDE4B, BAG3, SORBS2, LMOD1, PALLD, DES, CSRP2, MYL9, CRYAB, CALD1, TPM2, PDLIM1, TNNC1
Z disc	GO:0,030,018	3.04E-07	HOMER1, FHOD3, LDB3, FHL5, CASQ2, PDE4B, BAG3, SORBS2, PALLD, DES, CSRP2, MYL9, CRYAB, PDLIM1
Site of polarized growth	GO:0,030,427	3.07E-07	PCDH9, FGF13, SNAP25, ELAVL4, STMN2, MAPT, KIF5C, FRYL, GPM6A, NDRG2, OLFM1, PALLD, NEFL, FEZ1, EPS8, FRY
Actin filament bundle	GO:0,032,432	4.74E-07	LDB3, ACTA2, LPP, BAG3, PALLD, MYL9, CRYAB, LIMCH1, MYLK, PLS3, PDLIM1
I band	GO:0,031,674	9.22E-07	HOMER1, FHOD3, LDB3, FHL5, CASQ2, PDE4B, BAG3, SORBS2, PALLD, DES, CSRP2, MYL9, CRYAB, PDLIM1
Myofibril	GO:0,030,016	1.01E-06	HOMER1, FHOD3, LDB3, FHL5, CASQ2, PDE4B, BAG3, SORBS2, LMOD1, PALLD, DES, CSRP2, MYL9, CRYAB, CALD1, TPM2, PDLIM1, TNNC1
Sarcomere	GO:0,030,017	1.34E-06	HOMER1, FHOD3, LDB3, FHL5, CASQ2, PDE4B, BAG3, SORBS2, LMOD1, PALLD, DES, CSRP2, MYL9, CRYAB, TPM2, PDLIM1, TNNC1
Blood microparticle	GO:0,072,562	1.81E-06	APOA1, IGKV1-17, ACTG2, IGLC1, IGHM, STOM, IGHG1, HBA1, HBB, AGT, IGKC, SERPINA3, HSPA1A, CFH
Distal axon	GO:0,150,034	2.84E-06	PCDH9, TNN, FGF13, SNAP25, ELAVL4, ADRA2A, STMN2, CALCA, MAPT, KIF5C, GPM6A, PRKCB, NDRG2, AAK, OLFM1, PALLD, NEFL, FEZ1, EPS8
*Molecular function*			
Extracellular matrix structural constituent	GO:0,030,021	7.84E-05	ACAN, DCN, PRELP, LUM, ASPN
Integrin binding	GO:0,005,178	3.28E-08	TSPAN8, TNN, ITGB5, COL4A3, ITGA6, ADAM22, ICAM2, ICAM1, FGF1, VWF, THBS1, THBS4, CX3CL1, GFAP, CXCL12, FBLN1, IGF2
RAGE receptor binding	GO:0,050,786	9.24E-07	S100A9, S100A4, S100A12, S100A8, S100B
Receptor ligand activity	GO:0,048,018	1.78E-06	CCL19, CSPG5, GRP, FGF13, APOA1, CALCA, SEMA3G, EDN1, INHBA, CXCL2, CXCL8, HBEGF, CCL14, PPBP, IL6, FGF1, IL1RN, NAMPT, THBS4, STC1, CX3CL1, AGT, CCL2, CXCL12, SCG2, ADM, BMP5, IGF2
Signaling receptor activator activity	GO:0,030,546	2.34E-06	CCL19, CSPG5, GRP, FGF13, APOA1, CALCA, SEMA3G, EDN1, INHBA, CXCL2, CXCL8, HBEGF, CCL14, PPBP, IL6, FGF1, IL1RN, NAMPT, THBS4, STC1, CX3CL1, AGT, CCL2, CXCL12, SCG2, ADM, BMP5, IGF2
G protein-coupled receptor binding	GO:0,001,664	3.28E-06	S1PR1, HOMER1, CCL19, ADRA2A, CALCA, EDN1, CXCL2, CXCL8, CCL14, PPBP, NES, NEDD4, TAC1, GPRC5B, CX3CL1, AGT, CCL2, CXCL12, HSPA1A, ADM
Chemokine activity	GO:0,008,009	7.53E-06	CCL19, CXCL2, CXCL8, CCL14, PPBP, CX3CL1, CCL2, CXCL12
Calcium-dependent protein binding	GO:0,048,306	1.42E-05	SNAP25, STMN2, CASQ2, S100A9, S100A4, SYT1, S100A12, S100A8, S100B, TNNC1
DNA-binding transcription activator activity, RNA polymerase II-specific	GO:0,001,228	1.69E-05	SOX17, ERG, REL, ESR1, MECOM, FOSL1, MAFF, JUN, KLF6, CEBPB, TCF4, EGR2, NR4A3, NFIB, NR3C1, FOSL2, KLF10, NFATC1, SOX9, NR4A2, ATF3, EGR1, NR4A1, FOSB, FOS
Chemokine receptor binding	GO:0,042,379	1.72E-05	CCL19, CXCL2, CXCL8, CCL14, PPBP, NES, CX3CL1, CCL2, CXCL12
*Biological process*			
Leukocyte migration	GO:0,050,900	1.37E-12	S1PR1, CHGA, CCL19, SELE, GPR183, CALCA, EDN1, PECAM1, CXCL2, CXCL8, CD34, CCL14, ITGA6, PPBP, IL6, S100A9, ICAM1, S100A12, FLT1, C5AR1, PDE4B, THBS1, S100A8, CD200, THBS4, CXCR4, IL1R1, CX3CL1, CCL2, EPS8, CXCL12, CH25H, SCG2, BMP5
Detoxification of copper ion	GO:0,010,273	3.12E-12	MT3, MT1G, MT1E, MT1M, MT2A, MT1HL1, MT1X, MT1F, MT1H
Stress response to copper ion	GO:1,990,169	3.12E-12	MT3, MT1G, MT1E, MT1M, MT2A, MT1HL1, MT1X, MT1F, MT1H
Cellular divalent inorganic cation homeostasis	GO:0,072,503	4.72E-12	S1PR1, CCL19, PLN, CALCA, CASQ2, EDN1, MT3, RAMP3, ESR1, PRKCB, CCL14, ITPR1, S100A9, MT1G, C5AR1, CD24, MT1E, MT1M, MT2A, MT1HL1, YWHAE, S100A8, MT1X, MT1F, BNIP3, CD55, STC1, TAC1, MT1H, CXCR4, CX3CL1, AGT, CXCL12, CAV1, ATP1A2, PDGFRA, ADM, PTGDR
Cell chemotaxis	GO:0,060,326	4.81E-12	S1PR1, CHGA, CCL19, GPR183, CALCA, EDN1, CXCL2, CXCL8, HBEGF, CCL14, PPBP, IL6, S100A9, FGF1, S100A12, FLT1, C5AR1, PDE4B, FGFR1, THBS1, S100A8, THBS4, CXCR4, CX3CL1, CCL2, CXCL12, CH25H, PDGFRA, SCG2, NR4A1
Muscle contraction	GO:0,006,936	1.29E-11	HOMER1, MYH11, CNN1, CHGA, PLN, EHD3, SSPN, FGF13, PTGS2, ADRA2A, CALCA, CASQ2, EDN1, ACTA2, PDE4B, ABAT, LMOD1, DES, STC1, CXCR4, AGT, MYL9, RGS2, CRYAB, CALD1, CAV1, ATP1A2, MYLK, TPM2, NR4A1, TNNC1
Muscle system process	GO:0,003,012	2.38E-11	HOMER1, MYH11, CNN1, CHGA, PLN, EHD3, SSPN, FGF13, PTGS2, ADRA2A, CALCA, CASQ2, EDN1, RGS4, ACTA2, PDE4B, ABAT, SORBS2, LMOD1, DES, STC1, CXCR4, NR4A3, AGT, MYL9, LMCD1, RGS2, CRYAB, CALD1, CAV1, ATP1A2, MYLK, TPM2, NR4A1, TNNC1
Muscle tissue development	GO:0,060,537	2.82E-11	S1PR1, HOMER1, MYH11, PLN, FHOD3, ADAMTS9, EDN1, CYP26B1, RGS4, MAFF, NDRG4, SAP30, SORBS2, ZFAND5, EGR2, ID2, CSRP2, AGT, RGS2, CAV1, MYLK, PDGFRA, ALDH1A2, SOX9, ATF3, EGR1, NR4A1, SIK1, RBP4, BMP5, TNNC1, FOS, IGF2
Detoxification of inorganic compound	GO:0,061,687	2.87E-11	MT3, MT1G, MT1E, MT1M, MT2A, MT1HL1, MT1X, MT1F, MT1H
Leukocyte chemotaxis	GO:0,030,595	3.07E-11	S1PR1, CHGA, CCL19, GPR183, CALCA, EDN1, CXCL2, CXCL8, CCL14, PPBP, IL6, S100A9, S100A12, FLT1, C5AR1, PDE4B, THBS1, S100A8, THBS4, CXCR4, CX3CL1, CCL2, CXCL12, CH25H, SCG2

Category refers to the GO functional categories

**Table 2:** Pathway enrichment analysis for the differentially expressed genes (DEGs)

Term	Category	*P* value	Genes name
PI3K-Akt signaling pathway	hsa04151	7.79E-05	TNN, ITGB5, COL4A3, TNC, ITGA6, IL6, FGF1, ANGPT1, FLT1, VWF, FGFR1, THBS1, YWHAE, LAMA2, THBS4, COL2A1, CCND1, PDGFRA, COL9A3, NR4A1, DDIT4, COL4A2, IGF2
Focal adhesion	hsa04510	9.20E-08	TNN, ITGB5, COL4A3, PRKCB, TNC, ITGA6, PPP1CB, JUN, FLT1, VWF, THBS1, LAMA2, THBS4, MYL9, COL2A1, CAV1, CCND1, MYLK, PDGFRA, COL9A3, COL4A2
MAPK signaling pathway	hsa04010	0.000962	MAPT, PRKCB, MECOM, FGF1, ANGPT1, JUN, FLT1, GADD45B, DUSP2, FGFR1, IL1R1, NFATC1, PDGFRA, HSPA1A, DUSP5, NR4A1, FOS, IGF2
Proteoglycans in cancer	hsa05205	0.000127	ITGB5, ESR1, PRKCB, HBEGF, CD44, ITPR1, PPP1CB, FGFR1, THBS1, PLAUR, DCN, FZD2, CAV1, CCND1, LUM, IGF2
AGE-RAGE signaling pathway in diabetic complications	hsa04933	6.00E-08	SELE, EDN1, COL4A3, PRKCB, CXCL8, THBD, IL6, ICAM1, JUN, AGT, CCL2, NFATC1, CCND1, EGR1, COL4A2
IL-17 signaling pathway	hsa04657	1.84E-07	PTGS2, CXCL2, CXCL8, FOSL1, IL6, S100A9, JUN, S100A8, CEBPB, TNFAIP3, NFKBIA, CCL2, FOSB, FOS
Vascular smooth muscle contraction	hsa04270	1.39E-05	MYH11, CALCA, EDN1, RAMP3, PRKCB, ITPR1, ACTG2, PPP1CB, ACTA2, AGT, MYL9, CALD1, MYLK, ADM
Lipid and atherosclerosis	hsa05417	0.001987	SELE, APOA1, CXCL2, CXCL8, ITPR1, IL6, ICAM1, JUN, LY96, NFKBIA, CCL2, NFATC1, HSPA1A, FOS
ECM-receptor interaction	hsa04512	5.63E-07	TNN, ITGB5, COL4A3, TNC, CD44, ITGA6, VWF, THBS1, LAMA2, THBS4, COL2A1, COL9A3, COL4A2
TNF signaling pathway	hsa04668	9.00E-06	SELE, PTGS2, EDN1, CXCL2, IL6, ICAM1, JUN, CEBPB, TNFAIP3, NFKBIA, CX3CL1, CCL2, FOS
NOD-like receptor signaling pathway	hsa04621	0.001382	CXCL2, CXCL8, GBP2, ITPR1, IL6, JUN, GABARAPL1, YWHAE, NAMPT, TNFAIP3, NFKBIA, CCL2, IFI16
NF-kappa B signaling pathway	hsa04064	2.13E-05	CCL19, PTGS2, PRKCB, CXCL2, CXCL8, ICAM1, GADD45B, LY96, TNFAIP3, NFKBIA, IL1R1, CXCL12
Fluid shear stress and atherosclerosis	hsa05418	0.000358	SELE, IL1R2, EDN1, PECAM1, THBD, PLAT, ICAM1, JUN, IL1R1, CCL2, CAV1, FOS
Malaria	hsa05144	6.34E-08	ACKR1, SELE, PECAM1, CXCL8, IL6, ICAM1, THBS1, THBS4, HBA1, HBB, CCL2
Rheumatoid arthritis	hsa05323	0.00019	CXCL2, CXCL8, IL6, ICAM1, ANGPT1, JUN, FLT1, CCL2, CXCL12, FOS
Viral protein interaction with cytokine and cytokine receptor	hsa04061	0.000345	CCL19, CXCL2, CXCL8, CCL14, PPBP, IL6, CXCR4, CX3CL1, CCL2, CXCL12
Mineral absorption	hsa04978	2.87E-05	MT1G, MT1E, MT1M, MT2A, MT1HL1, MT1X, MT1F, MT1H, ATP1A2
Hypertrophic cardiomyopathy	hsa05410	0.00068	ITGB5, EDN1, ITGA6, IL6, LAMA2, DES, AGT, TPM2, TNNC1
Amoebiasis	hsa05146	0.00167	IL1R2, COL4A3, PRKCB, CXCL2, CXCL8, IL6, LAMA2, IL1R1, COL4A2
African trypanosomiasis	hsa05143	4.87E-05	SELE, APOA1, PRKCB, IL6, ICAM1, HBA1, HBB

### Construction of PPI network and selection of the Hub gene

**Fig. 3:**
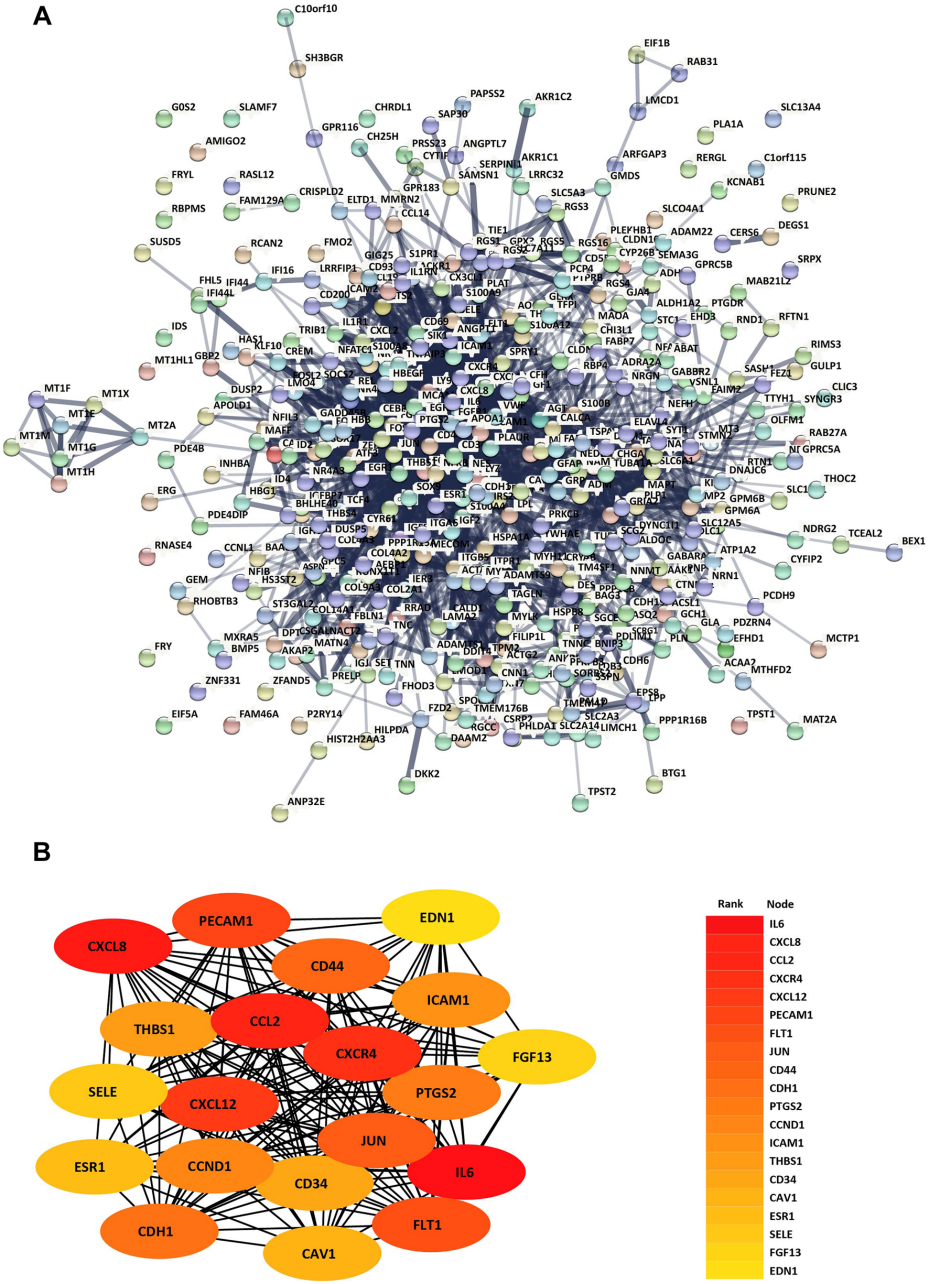
PPI network and top module of 420 DEGs. **A** PPI network of DEGs in light blue and top one module in orange. **B** Top 20 hub genes

To demonstrate the potential PPI correlations, the PPI network of the 420 DEGs was constructed with the STRING and visualized with Cytoscape software. There were 404 nodes and 2250 edges in the PPI network (Fig. 3). Among these, top 20 hug gene were identified with CytoHubba (Cytoscape plugin), including C–X–C motif chemokine ligand 8 (CXCL8), Jun proto-oncogene (JUN), Interleukin 6 (IL6), C–X–C Motif Chemokine Receptor 4 (CXCR4), C–X–C motif chemokine ligand 12 (CXCL12), C–C motif chemokine ligand 2 (CCL2), Platelet And Endothelial Cell Adhesion Molecule 1 (PECAM1), Fms Related Receptor Tyrosine Kinase 1 (FLT1), CD44, Cadherin 1 (CDH1), PTGS2 prostaglandin-endoperoxide synthase 2 (PTGS2), Cyclin D1 (CCND1), Intercellular Adhesion Molecule 1 (ICAM1), Thrombospondin 1 (THBS1), CD34, Caveolin 1 (CAV1), Estrogen Receptor 1 (ESR1), Selectin E (SELE), Fibroblast Growth Factor 13 (FGF13), endothelin 1 (EDN1).

### Analyzes of immune infiltration in meningioma tumors

**Fig. 4:**
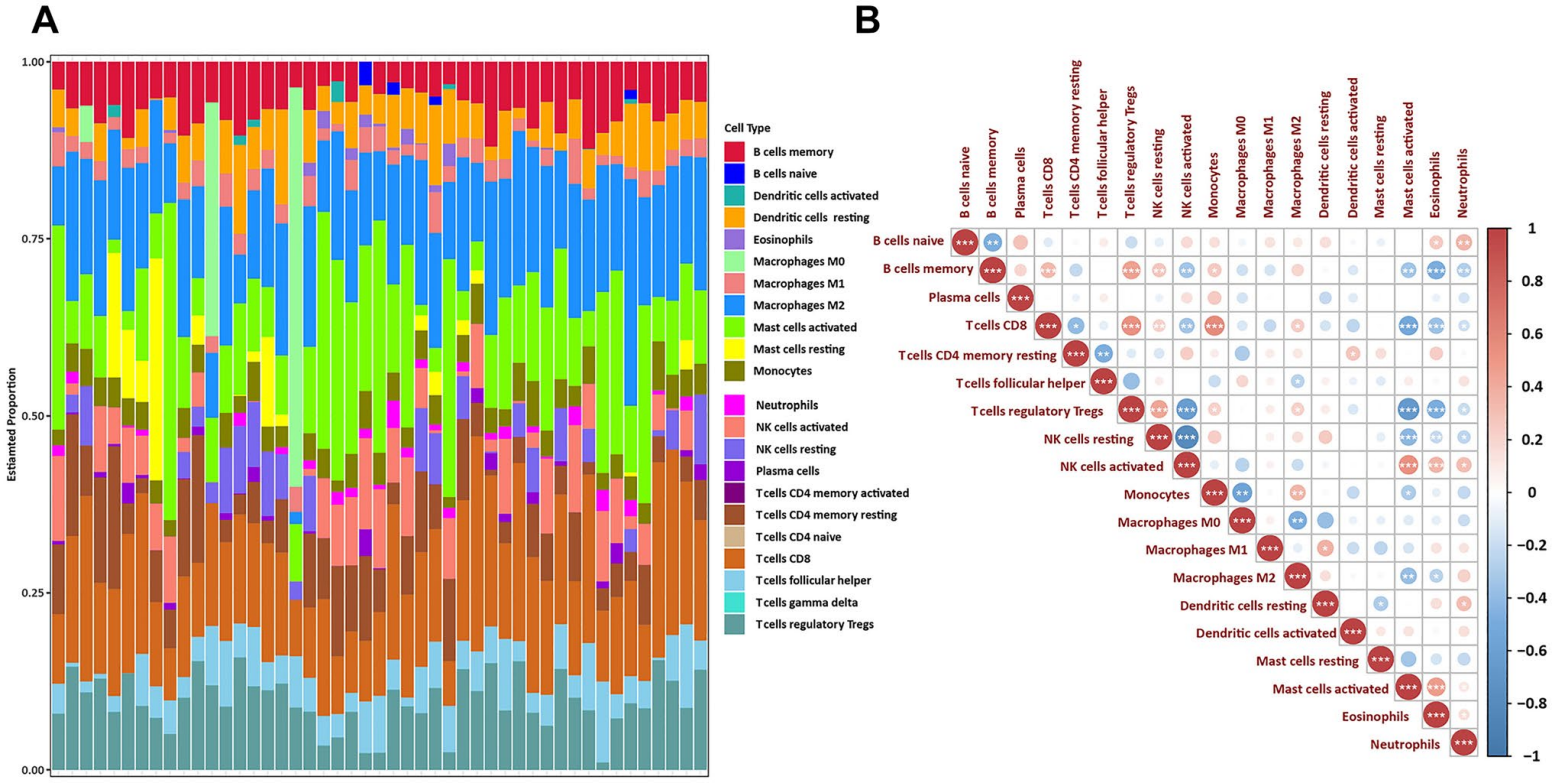
Visualization of immune cell infiltration. **A** The relative percentage of 22 kinds of immune cells. **B** Interaction of 22 immune cells, as well as immune and stromal scores. Orange represents positive correlation and blue represents negative correlation. The darker the color indicating the greater the greater correlation coefficient. *P* < 0.05 indicated statistically significant. Note: ∗ *P* value < 0.05, ∗  ∗ *P* value < 0.01, ∗  ∗  ∗ *P* value < 0.001, ∗  ∗  ∗  ∗ *P* value < 0.0001, *ns* not significant

**Fig. 5:**
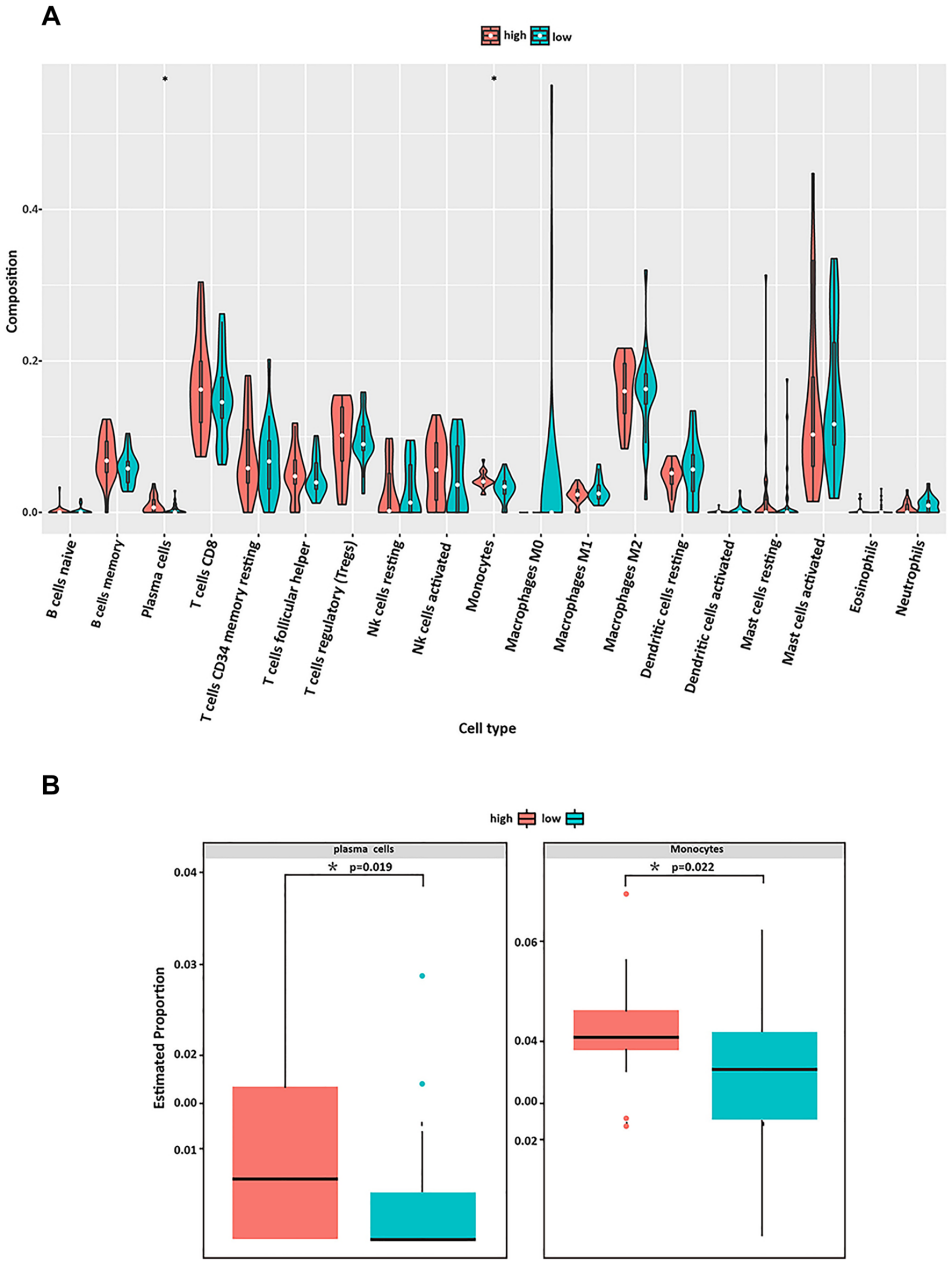
Evaluation of immune cell infiltration. **A** The violin plot image of immune cells in meningioma tissue in PD-L1 high expression group and low expression group. **B** Estimated proportion of plasma cells and monocyte infiltration in PD-L1 high expression group and low expression group. *P* < 0.05 indicated statistically significant

The immune infiltration profiles in normal meninges and meningioma groups were explored with the 22 subpopulations of immune cells. The percentage of the 22 types of immune cells was visually displayed in Fig. 4A. The Pearson correlations among the 22 immune cell types’ infiltrations and the immune scores in meningioma patients showed that T cell CD8 were positively correlated to monocytes and negatively correlated with Mast cells activated; B cell memory were positively correlated with T cells regulatory Tregs, and negatively correlated with Eosinophils (Fig. 4B). CIBERPORT analysis showed that the infiltration levels of plasma cells (*P* = 0.019) and monocyte infiltration (*P* = 0.022) was significantly increased in the PD-L1 high meningioma group (Fig. 5).

### Correlation analysis of hub-gene expression with PD-L1

**Fig. 6:**
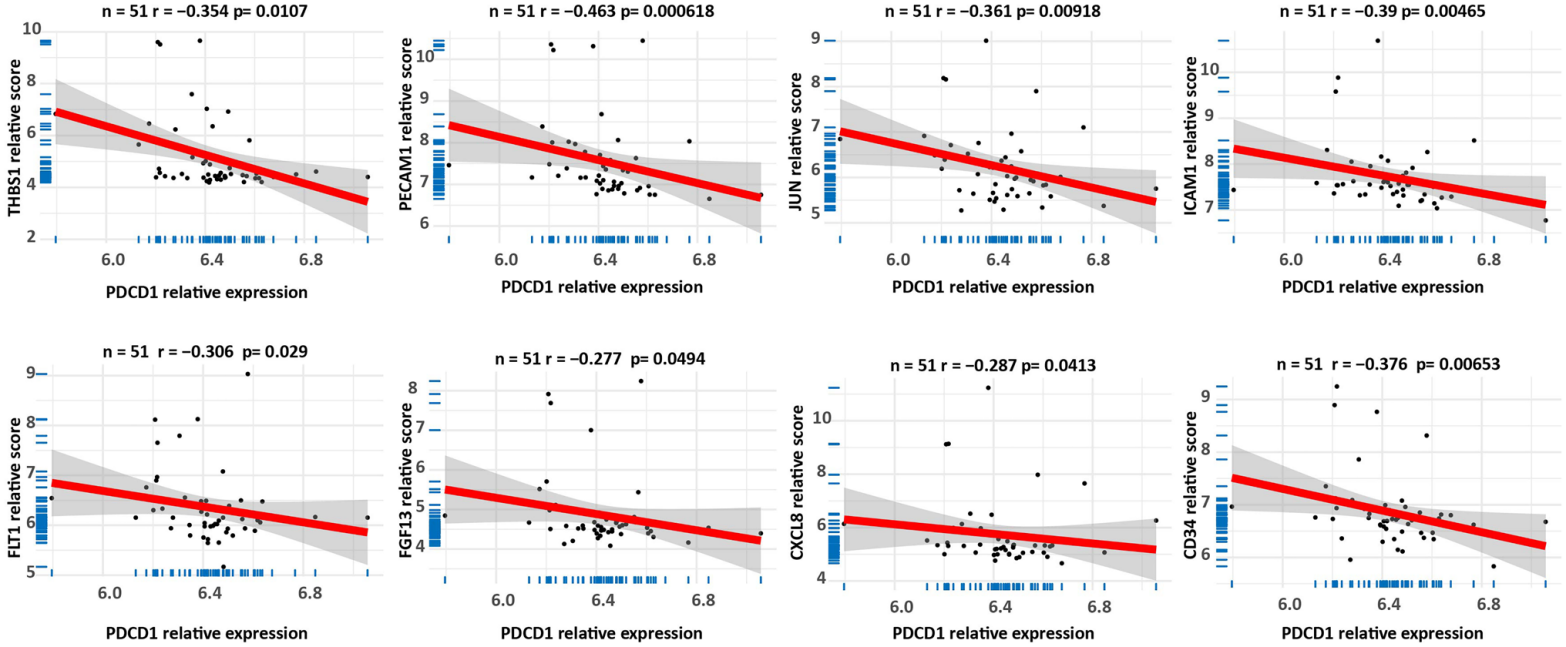
Correlation between PD-L1 and hub genes

To investigate the regulatory mechanisms of PD-L1 in meningioma, we further analyzed the correlation between hub-gene expression and PD-L1. There are 8 DEGs among the 20 hub genes showed negative correlation with PD-L1, including PECAM1 (*r* = − 0.463, *P* = 0.000618), JUN (*r* = − 0.361, *P* = 0.00618), CD34 (*r* = − 0.376, *P* = 0.00653), FLT1 (*r* = − 0.306, *P* = 0.029), CXCL8 (*r* = − 0.287, *P* = 0.0413), ICAM1 (*r* = − 0.39, *p* = 0.00465), THBS1 (*r* = − 0.354, *P* = 0.0107), and FGF13 (*r* = − 0.277, *P* = 0.0494) (Fig. 6).

### TF-miRNA coregulatory network analysis

**Fig. 7:**
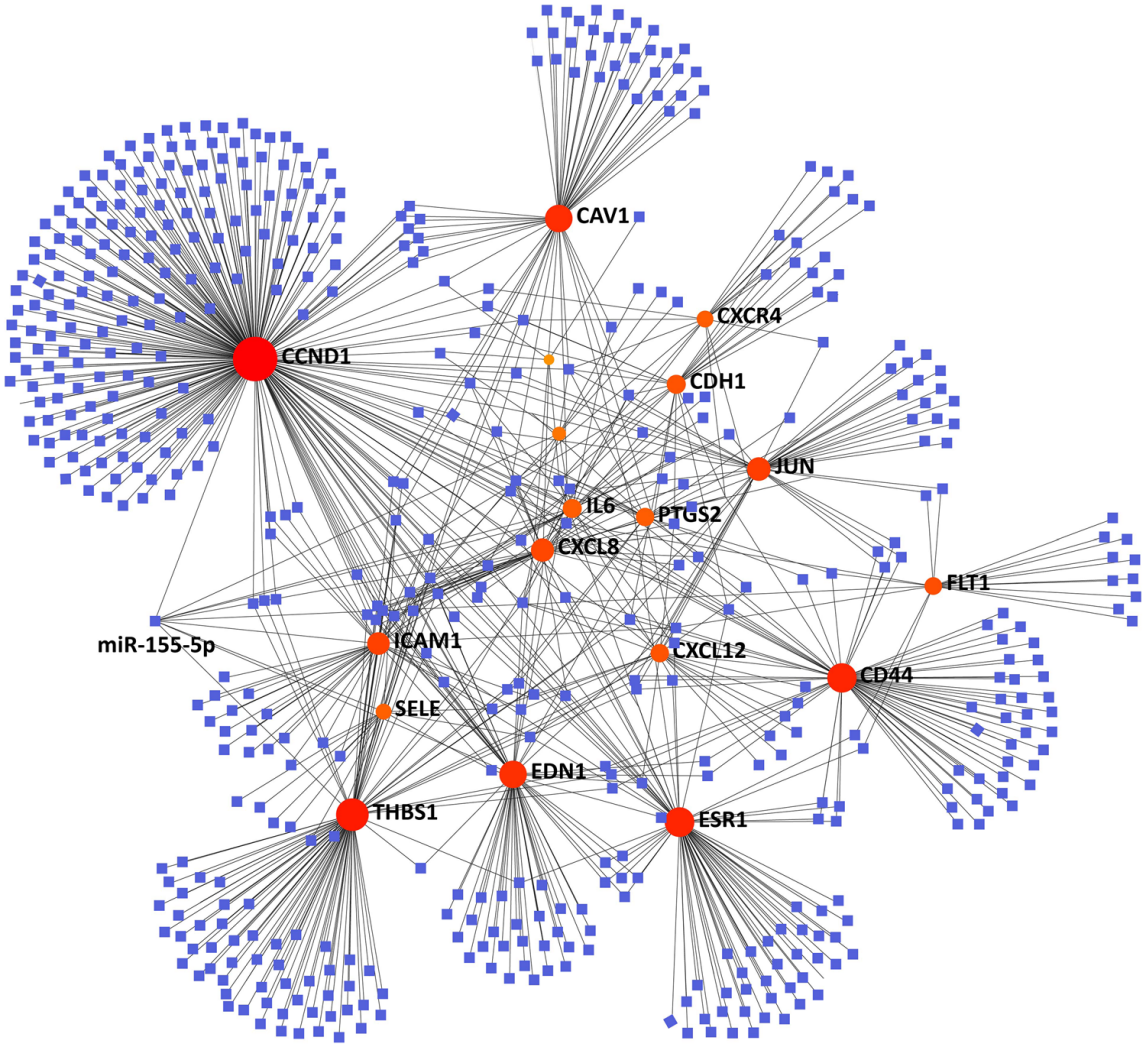
The network presents the TF-miRNA coregulatory network. The pink circle means the selected DEGs and the blue square means the miRNAs

The analysis of the TF-miRNA coregulatory network delivers miRNAs and TFs interaction with the common DEGs. This interaction can be the reason for regulating the expression of the DEGs. To analyze miRNA, TF interactions, TF-miRNA coregulatory network is generated using NetworkAnalyst. The network created for TF-miRNA coregulatory network comprises 53 nodes and 204 edges. Figure 7 dispenses TF-miRNA coregulatory network.

## Discussion

Meningiomas are the most common primary tumor happed CNS. The clinical observation showed that meningiomas are frequently result in focal neurological deficits, seizures [1, 21]. For the management of meningiomas, maximal safe surgical resection remains the standard of treatment. However, the ability to achieve complete resection may be limited by a number of factors, including tumor location; involvement of nearby dural venous sinuses, arteries, cranial nerves, and brain invasion into eloquent tissue [22, 22]. Recently, meningiomas are no longer considered as benign diseases. Molecular characterization of meningioma could provide potential therapeutic targets especially for meningioma recurrence.

Derived from the meninge, meningioma is a unique tumor with potential for mesenchymal and epithelial differentiating. And one of the major mesenchymal functions of meningioma cells is their ability to elaborate extracellular matrix proteins [23, 24]. One previous immunohistochemical study showed that fibronectin, galectin-3, matrix metalloproteinase 2, matrix metalloproteinase 9, and collagen IV were highly expressed in meningioma tissues [25]. In our study, we compared the gene expression profiles of meningioma tumor and normal meninge. The results observed in this study are partly consistent with previous studies. We found that the expression of COL2A1, and COL9A3 were significantly upregulated in meningioma tissues, and the expression of COL14A1, COL4A3, and COL4A2 was significantly down-regulated. As previous studies demonstrated that the extracellular matrix (ECM) proteins are involved in invasion, edema formation, and metastasis in various tumors [26, 27]. Thus, we proposed that the differentially expressed collagens found in this study might be potential targets of future therapy. Moreover, the DEGs were mostly enriched in KEGG pathways such as PI3K-Akt signaling pathway, focal adhesion, MAPK signaling pathway, and proteoglycans in cancer. Notably, it has been reported that both MAPK and PI3K/Akt pathways are activated in benign and malignant meningiomas [28]. Activation of PI3K/Akt signaling might be responsive to the aggressive behavior of malignant meningiomas, whereas MAPK activation contributes to their proliferation and apoptosis [29]. And co-targeting PI3K/Akt/mTOR and MAPK pathways improved cell proliferation inhibition in comparison to the target of each pathway alone [30]. In this study, we found that 2 hub genes, FLT1, and THBS1, participated in both the PI3K-Akt signaling pathway and MAPK signaling pathway, which deserves further investigation. Here, we also claimed that further understanding of the signaling pathways involved in meningioma tumorigenesis will lead to better treatment modalities in the future.

Emerging evidence has shown that immunotherapy, particularly checkpoint inhibition, could improve survival in some solid tumors such as lung cancer and melanoma patients [31–33]. Recent studies have investigated the interactions between meningiomas and the immune system. And several potential immunotherapeutic targets including PD-L1, NY-ESO-1, B7-H3, and CTLA-4 showed their potential for the anti-tumor therapies in clinical settings [34, 35]. The immune infiltration of meningiomas and their characterization have been well documented in the literatures. Previous study has detected PD-L1 expression in meningiomas in both tumor and immune cells and observed intra and inter tumoral heterogeneity [36]. And overexpression of PD-L1 described as an independent prognostic marker for worse recurrence free survival in meningioma [34]. The expression of these proteins has been associated with tumor progression, recurrence, and poor survival outcomes [37]. In consistent with previous studies, our result showed that immune cell infiltrates of meningiomas include variable numbers of T cells, B cells, plasma cells, monocytes, and macrophages [38, 39]. Moreover, our results also show that indicated that in the PD-L1 high expression group the infiltration of plasma cell and monocytes were increased. Evidence have reported that PD-L1 could expression on the tumor-infiltrating non-malignant cells such as plasma cell and monocytes [40, 41]. However, the interaction between the tumors and immune cells in meningioma is not yet fully characterized which might paly crucial roles in PD-L1 Blockade Therapy.

Further, our result showed that the expression of several hug genes was negatively correlated with PD-L1, including FLT1, CXCL8, JUN, THBS1, FECAM1, CD34, and FGF13. And TF-miRNA coregulatory network analysis showed that miR‐155‐5p with the highest network betweenness could regulate their expression. As mentioned above, those DEGs were negatively correlated with PD-L1. Here, our results suggested the potential use of miR‐155‐5p for anti-PD‐L1 therapy for meningiomas. It has been reported that miR‐155‐5p is a key oncogenic microRNA that maintains immune homeostasis and mediates cross‐talk between inflammation and tumorigenesis [42]. Previous studies have indicated that miR‐155‐5p is highly expressed in many cancers, such as lung cancer, breast cancer, colon cancer, lymphoma, and other tumors [43, 44]. Currently, several studies have investigated the relationship between miR‐155‐5p and PD‐L1 in cancer. Evidence from lung cancers demonstrated that miR‐155‐5p could suppress the expression of PD‐L1 [45]. However, the effect of miR‐155‐5p on PD‐L1 in meningiomas remain underrepresentative. Thus, a deeper investigation of the potential interaction between miR‐155 and PD‐L1 provide new insights into the immune response of meningioma.

There were limitations to our study, though. First, in this study we performed several bioinformatics analyses based one the published data without experimental verification. Second, we only analyzed the correlation between the top 20 hub genes with PD-L1 positive expression. It would also be important to investigate the correlations with another immune checkpoint inhibitors such as NY-ESO-1, B7-H3, and CTLA-4. Moreover, clinical studies with larger cohorts are needed to validate our results in the future work.

## Conclusion

In this study, we compared the gene expression pattern of meningioma and normal meninge tissue with a series of bioinformatics analyses. Among the hub genes, FLT1, CXCL8, JUN, THBS1, FECAM1, CD34, and FGF13 were negatively correlated with PD-L1. And the expression of those genes was co-regulated by miR‐155‐5p. Our finding suggested a potential use of miR‐155‐5p for anti-PD‐L1 therapy of meningioma, which deserves further investigation.

## Supplementary Information

Below is the link to the electronic supplementary material.

Supplementary file1 (CSV 32 kb) The summary of 15 up-regulated and 405 down-regulated DEGs

## Data Availability

The datasets are available in the National Center of Biotechnology Information (NCBI) GEO (https://www.ncbi.nlm.nih.gov/geo/). one datasets GSE43290 was used in the present study.

## Abbreviations

GEO: Gene expression omnibus
DEGs: Differentially expressed genes
PPI: Protein–protein interaction
TF: Transcription factor
WHO: The World Health Organization
NF2: Neurofibromatosis type 2
FAK: Focal adhesion kinase
GO: Gene ontology
CCs: Cellular components
BPs: Biological processes
MFs: Molecular functions
CXCL8: C–X–C motif chemokine ligand 8
JUN: Jun proto-oncogene
IL6: Interleukin 6
CXCR4: C–X–C motif chemokine receptor 4
CXCL12: C–X–C motif chemokine ligand 12
CCL2: C–C motif chemokine ligand 2
PECAM1: Platelet and endothelial cell adhesion molecule 1
FLT1: Fms related receptor tyrosine kinase 1
CDH1: Cadherin 1
PTGS2: PTGS2 prostaglandin-endoperoxide synthase 2
CCND1: Cyclin D1
ICAM1: Intercellular adhesion molecule 1
THBS1: Thrombospondin 1
CAV1: Caveolin 1
ESR1: Estrogen receptor 1
SELE: Selectin E
FGF13: Fibroblast growth factor 13
EDN1: Endothelin 1
ECM: Extracellular matrix
PD-L1: Programmed death ligand-1
PD-L2: Programmed death ligand-2
